# Effects of continuity of care on hospitalizations and healthcare costs in older adults with dementia

**DOI:** 10.1186/s12877-022-03407-7

**Published:** 2022-09-02

**Authors:** Yung-Hsiang Chao, Wen-Yen Huang, Chia-Hong Tang, Yu-An Pan, Jeng-Yuan Chiou, Li-Jung Elizabeth Ku, James Cheng-Chung Wei

**Affiliations:** 1 Institute of Medicine, Chung Shan Medical University, Taichung, Taiwan; 2 Department of Public Health, College of Medicine, National Cheng Kung University, No.1, University Road, Tainan City, 701 Taiwan; 3 Department of Psychiatric, Tainan Hospital, Ministry of Health and Welfare, Tainan City, Taiwan; 4 Department of Sociomedical Sciences, Mailman School of Public Health, Columbia University, New York, NY USA; 5 School of Health Policy and Management, Chung Shan Medical University, Taichung, Taiwan; 6 Graduate Institute of Integrated Medicine, China Medical University, Taichung, Taiwan; 7 Department of Allergy, Immunology & Rheumatology, Chung Shan Medical University Hospital, Taichung, Taiwan

**Keywords:** Dementia, Ambulatory care, Hospitalization, Health care costs, Continuity of patient care

## Abstract

**Introduction:**

People with dementia have high rates of hospitalization, and a share of these hospitalizations might be avoidable with appropriate ambulatory care**,** also known as potentially preventable hospitalization (PAH). This study investigates the associations between continuity of care and healthcare outcomes in the following year, including all-cause hospitalization, PAHs, and healthcare costs in patients with dementia.

**Methods:**

This is a longitudinal retrospective cohort study of 69,658 patients with dementia obtained from the Taiwan National Health Insurance Research Database. The Continuity of Care Index (COCI) was calculated to measure the continuity of dementia-related visits across physicians. The PAHs were classified into five types as defined by the Medicare Ambulatory Care Indicators for the Elderly (MACIEs). Logistic regression models were used to examine the effect of COCI on all-cause hospitalizations and PAHs, while generalized linear models were used to analyze the effect of COCI on outpatient, hospitalization, and total healthcare costs.

**Results:**

The high COCI group was significantly associated with a lower likelihood of all-cause hospitalization than the low COCI group (OR = 0.848, 95%CI: 0.821–0.875). The COCI had no significant effect on PAHs but was associated with lower outpatient costs (exp(β) = 0.960, 95%CI: 0.941 ~ 0.979), hospitalization costs (exp(β) = 0.663, 95%CI: 0.614 ~ 0.717), total healthcare costs (exp(β) = 0.962, 95%CI: 0.945–0.980).

**Conclusion:**

Improving continuity of care for dementia-related outpatient visits is recommended to reduce hospitalization and healthcare costs, although there was no statistically significant effect of continuity of care found on PAHs.

## Introduction

Worldwide, around 50 million people have dementia, and this number is projected to increase by 64% in the next 10 years [1]. The aging of the dementia population has led to an increase in the number of families facing unique challenges to the healthcare system [2]. The WHO listed dementia as a global public health priority in 2012, calling for immediate action to promote dementia as a priority for national public health and social care systems [3]. Numerous studies have shown that people with dementia are more likely to have hospital admissions compared to people of similar age and gender [4], and longer hospital stays [5]. Higher medical costs related to dementia are mainly driven by a significantly higher risk of hospitalization associated with dementia compared to those without [5, 6]. Since a previous study has shown that the higher Medicare expenditures associated with the diagnosis of dementia were primarily due to more hospitalizations [7], the growing population of older adults with dementia also plays an important role in Taiwan’s healthcare system [8].

Individuals with dementia have high rates of hospitalization [6], and a share of these hospitalizations might be avoidable with appropriate ambulatory care [9], also known as potentially avoidable hospitalization (PAH) [10]. PAH refers to hospital admissions preventable by early intervention, and with good ambulatory care to avoid severe disease [11]. PAH is frequently a result of ambulatory care-sensitive conditions (ACSC; e.g., angina, bacterial pneumonia, urinary tract infection, dehydration, diabetes, hypertension), so hospitalization for ACSCs was deemed to be preventable with proactive ambulatory care [12]. PAH has been widely used as an indicator of access, quality, and primary health care performance, and overall health service [13, 14]. Researchers on PAH have chosen different operational measures for ACSCs, and two of the most common ACSC measures found in the literature include prevention quality indicators (PQIs) [15] and Medicare ambulatory care indicators for the elderly (the MACIEs) [16]. The PQIs are a set of measures consisting of 16 ACSCs, including pneumonia, chronic obstructive pulmonary disease (COPD), diabetes, hypertension, and urinary tract infection, and it was developed by the Agency for Healthcare Research and Quality to measure the occurrence of preventable hospitalizations [16]. On the other hand, the MACIEs were established as indicators to evaluate the quality of ambulatory care to the community-dwelling elderly and the set included five measures: (1) serious short-term complications of diabetes; (2) serious long-term complications of diabetes;(3) hypertension;(4) COPD/asthma;(5) heart failure [16]. For the current study, the MACIEs were selected as our preferred PAH measures of for the following two reasons. First, two previous studies have used the MACIEs to analyze PAH among patients with dementia [17, 18]. Secondly, the Taiwan’s Family Doctor Plan, a national program that pays office-based physicians to provide integrated care to their patients, have also selected the MACIEs as the Plan’s quality of care performance measures [19]. Therefore, this study selected the MACIEs for PAH outcomes in order to both compare with existing literature and for its relevance to Taiwan’s health policy.

Continuity of care (COC) is a major hallmark of health care delivery systems [20]. Continuity of care implies the degree to which patient visits are concentrated among medical providers [21]. A systematic review of 15 studies that examined the association between COC and ACSC hospitalizations concluded that increasing continuity in outpatient care is associated with a reduced likelihood of hospitalization for either all ACSCs or a specific ACSC [22] While the literature on the association between COC and PAHs have been consistent, there have only been a few studies that examined these associations focusing on the older adults with dementia and have reported mixed findings. One study in the older adults with dementia showed that improved ambulatory care might reduce the frequency of hospitalizations, which is of particular importance in cognitively impaired elderly due to increased complication rates [23]. Another study among community-dwelling older adults diagnosed with dementia found that lower continuity of care is associated with higher rates of hospitalization, emergency department visits, testing, and healthcare cost [21]. However, that same study found that better COC was associated with a lower rate of hospitalization for all causes, but not for ACSCs [21]. Since that was a cross-sectional study which could not ensure a temporal relationship, a recent cohort study in Canada showed that high primary care continuity might be an avenue for reducing potentially avoidable hospitalizations in community-dwelling persons with dementia on a population-wide scale [9]. Nevertheless, another large population-based observational study on older veterans with dementia in the US reached a different conclusion, showing that while better COC resulted in fewer hospitalizations, that effect was primarily due to less hospitalization for neuropsychiatric diseases/disorders and not hospitalization for ACSCs [24]. Given these mixed results, there remains a need to understand better the link between the continuity of care and potentially avoidable hospitalization in patients with dementia.

Better continuity of care is expected to improve patient outcomes and reduce healthcare costs [21, 25]. Most studies have reported that better COC was associated with favorable healthcare outcomes and reduced healthcare costs [21, 22]. For instance, a 2016 study on physician continuity and healthcare spending in older adults with dementia reported that total healthcare spending was higher with lower continuity (US$22,004 vs $24,371) after controlling for sociodemographic factors and comorbidity burden [21]. In a recent longitudinal study that examined the impact of COC on health care costs among older American veterans with dementia, it was shown that better COC resulted in lower institutional cost (acute inpatient, ED, and long-stay nursing home care), consistent with literature which showed that better COC was associated with a lower hospitalization rate and lower ED rate [26]. However, since the first study was a cross-sectional study and the second was based on a primarily male veteran sample, not representative of all patients with dementia, more evidence is needed to understand the potentially crucial role of COC in healthcare for persons with this complex, costly illness. Over 99% of individuals in Taiwan had been covered by the National Health Insurance (NHI) system since 1995, which allows for medical treatment and medical expenses for people with dementia [8]. Taiwan’s NHI program covered a wide range of health services, including outpatient, inpatient care, emergency care, and prescription drugs. Moreover, healthcare delivery in Taiwan focuses on specialist and hospital care without requirement for referrals [27]. The easy access to ambulatory care of offered by the NHI system has resulted in high COC between patients and physicians in Taiwan.

Therefore, the objectives of our study were to assess the effects of continuity of care on three sets of outcomes: (1) all-cause hospitalization (2) PAHs, and (3) healthcare expenditures in older adults with dementia. We hypothesized that higher continuity reduces all-cause hospitalizations and PAHs, and in turn, leads to lower costs of healthcare.

## Methods

### Data source and study sample

**Fig. 1:**
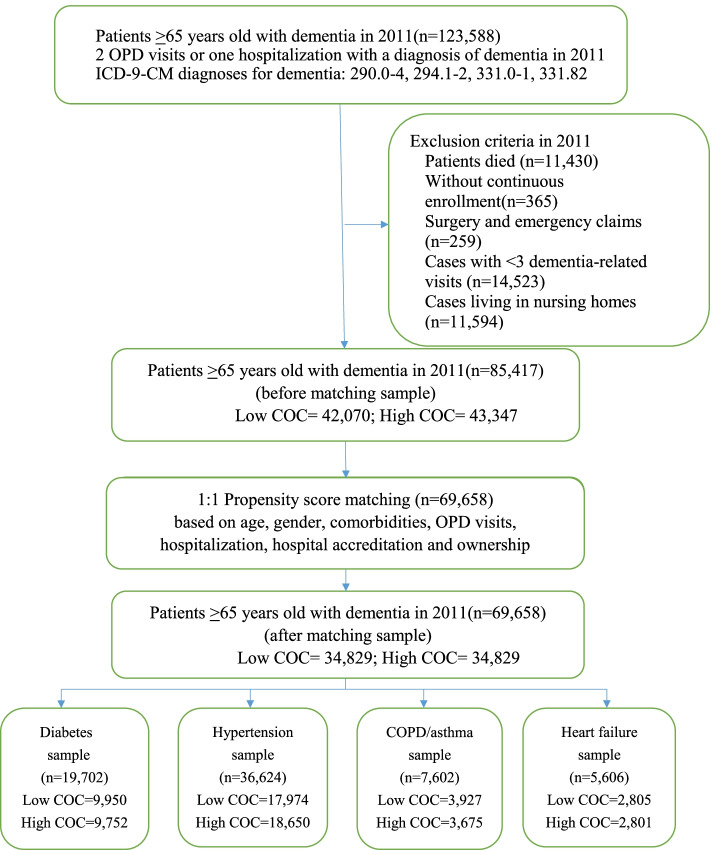
Sample selection flowchart

This is a longitudinal retrospective cohort study; the data were obtained from the Taiwan National Health Insurance Research Database (NHIRD). The flowchart of sample selection is depicted in Fig. 1. The study population comprised of patients with dementia aged 65 years and above having 2 outpatient (OPD) visits or 1 hospitalization in 2011 with a diagnosis of ICD-9-CM codes 290.0–4, 294.1–2, 331.0–1, and 331.82. To calculate the continuity of care index (COCI) for dementia-related visits across physicians in 2011, the following five exclusion criteria were applied: (1) individuals who died during the year 2011; (2) those without continuous insurance enrollment in 2011; (3) claims classified as outpatient surgery or emergency medical records (as they should not be included in COCI calculation); (4) cases with < 3 dementia-related outpatient visits in 2011; (5) cases living in nursing homes (since literature has shown that these residents were less likely to be hospitalized) [28]. The sample before matching comprised 85,417 older adults with dementia. Next, 1:1 propensity matching was conducted to form two groups of low COCI and high COCI patients, consisting of 34,829 people in each group. In addition to this primary sample, the second part of the study consisted of 4 disease-specific samples, including diabetes, hypertension, COPD/asthma, and heart failure. Each group included patients with the respective comorbidity, such as patients with dementia diagnosed with diabetes in 2011 as the diabetes sample. The sample size for the four PAH samples ranged from 5,606 for the smallest heart failure sample to 36,624 for the largest hypertension sample.

### Variables

Continuity of care was measured by the Bice-Boxerman Continuity of Care (BBC) index [29]. The continuity of care score is associated with various patient and physician characteristics [30]. Patients were divided into the high COCI group (COCI = 1) and low COCI group (COCI < 1) by the medium COCI score in 2011. 1:1 propensity score matching was performed to match the two groups of patients with dementia based on age, gender, comorbidities, outpatient visits or hospitalization in the baseline year (year 2011), hospital accreditation level, and ownership of hospital.

The Continuity of Care Index (COCI) was calculated to measure the continuity of care on patient visits across physicians for dementia-related outpatient visits in 2011. In this study, only visits with dementia ICD-9-CM codes (290, 294, and 331) were included for calculating dementia-related continuity of care. In Taiwan’s NHIRD, only the first three ICD-9-CM diagnostic codes were recorded from each outpatient visit. Therefore, if a physician put dementia among the top three ICD-9-CM codes, he or she presumably thought that the patient’s particular physician visit was addressing dementia-related issues, and thus capturing dementia-related COC. The COCI has a minimum score of 0 and a maximum score of 1, indicating the highest continuity of care.

The formula is as follows [31]:

$$\mathrm{COCI }= \left(\sum_{j=1}^M {\mathrm{n_{j}}}^{2}-\mathrm{N}\right) / \left(\mathrm{N }\left(\mathrm{N}-1\right)\right)$$

*N* = total number of visits across physicians in a year.

nj = number of visits that the patient has with the jth physician.

M = the number of physicians in a year.

The outcome variables examined in our study included all-cause hospitalization, five PAHs events, and medical costs. We used the admission date in the inpatient file to identify any all-cause hospitalization and we used discharge diagnosis codes to identify any PAHs in 2012. The PAHs were classified into five types: short-term complications of diabetes, long-term complications of diabetes, hypertension, COPD /asthma, and heart failure according to a list of diagnosis codes provided by MACIE (Medicare Ambulatory Care Indicators for the Elderly) [16]. As for the outcomes related to medical costs, the three measures included outpatient costs, hospitalization costs, and total healthcare costs.

In this study, we included three sets of control variables: (1) Characteristics of patients with dementia: gender, age, Charlson Comorbidity Index, (CCI) [32], low-income household status, residence location; (2) patients’ healthcare use: number of outpatient visits and any hospitalizations in the previous year (year 2010); (3) facility characteristics of the outpatient facility that the patient visited the most often in 2011: hospital accreditation level, hospital ownership. While we included patients’ healthcare use in year 2011 among variables used in propensity score matching, we included those variables in the year 2010 instead as control variables used in regression after matching. This choice was made to avoid controlling for the mediation effect of COC on hospitalization via baseline healthcare utilization, so that the healthcare use in previous year was controlled as a proxy for the patient’s disease severity.

### Statistical analysis

Descriptive statistics were used to examine the individual characteristics and healthcare use of patients with dementia according to high vs. low COCI groups in terms of frequency, percentages, means, and standard deviations. Chi-square tests and student’s t-tests were used to test for statistically significant differences between the high COCI and the low COCI groups before and after matching.

We used logistic regression models to examine the effect of COCI for patients with dementia on all-cause hospitalizations and PAHs in the following year. Three generalized linear models were used to analyze the impact of COCI on outpatient, hospitalization, and total healthcare costs in 2012. All statistical analyses were performed using SAS software, version 9.4.

## Results

### Characteristics of the study population

The basic characteristics of the study populations are shown in Table 1. Among our sample of 85,417 patients with dementia aged 65 or older, the overall mean of COCI was 0.74, the standard deviation was 0.3, and the median was 1. The patients were divided into the high COCI group (*n* = 43,347), and the other half into the low COCI group (*n* = 42,070) by the median value of 1. The two groups of low COCI and high COCI patients, each consisted of 34,829 people after 1:1 propensity matching. While there were significant differences between the two groups before matching, significant differences were found in fewer variables after matching: age, the distribution of CCI scores, and past hospital admissions. Despite there being a significant *p*-value in the t-tests for age, the difference in mean age, 80.3 in the low COCI group and 80.5 in the high COCI group, was negligible. However, previous year hospital admission rates remained different across the two COCI groups after matching (32.63% vs. 35.48%, *p*-value < 0.0001), and thus was included as a covariate in the subsequent regression model. Residence location was another covariate in the regression model since it was not included in the propensity matching, and therefore significant difference was found in the distribution of location both in the samples before and after matching.

**Table 1:** Characteristics of the study population

	Before matching (*n* = 85,417)			After matching (*n* = 69,658)		
	Low COCI^a^ (*n* = 42,070)	High COCI (*n* = 43,347)	*p*-value	Low COCI^a^ (*n* = 34,829)	High COCI (*n* = 34,829)	*p*-value
Patient characteristics						
Age (mean, SD)	80.8 ± 7	79.8 ± 7	< .0001***	80.3 ± 7	80.5 ± 7	0.0097**
Age (N, %)			< .0001***			0.0045**
65–69	2660 (6.32%)	3608 (8.32%)		2444 (7.02%)	2330 (6.69%)	
70–74	5648 (13.43%)	6999 (16.15%)		5075 (14.57%)	5025 (14.43%)	
75–79	8836 (21.00%)	9829 (22.68%)		7711 (22.14%)	7715 (22.15%)	
80–84	11,937 (28.37%)	11,563 (26.68%)		9871 (28.34%)	9614 (27.60%)	
≧85	12,989 (30.87%)	11,348 (26.18%)		9728 (27.93%)	10,145 (29.13%)	
Gender (N, %)			0.6791			0.2996
Male	17,844 (42.42%)	18,325 (42.28%)		14,603 (41.93%)	14,468 (41.54%)	
Female	24,226 (57.58%)	25,022 (57.72%)		20,226 (58.07%)	20,361 (58.46%)	
CCI score (mean, SD)	1.57 ± 1.63	1.48 ± 1.58	< .0001***	1.51 ± 1.59	1.50 ± 1.58	0.2512
CCI score (N, %)			< .0001***			0.0267**
0	12,498 (29.71%)	13,504 (31.15%)		10,864 (31.19%)	10,781 (30.95%)	
1	12,341 (29.33%)	13,255 (30.58%)		10,280 (29.52%)	10,599 (30.43%)	
≧2	17,231 (40.96%)	16,588 (38.27%)		13,685 (39.29%)	13,449 (38.61%)	
Low income household status (N, %)			< .0001***			0.5875
No	41,660 (99.03%)	43,018 (99.24%)		34,533 (99.15%)	34,546 (99.19%)	
Yes	410 (0.97%)	329 (0.76%)		296 (0.85%)	283 (0.81%)	
Residence location (N, %)^b^			< .0001***			< .0001***
Northern	17,646 (41.95%)	19,709 (45.47%)		14,696 (42.19%)	15,934 (45.75%)	
Central	11,027 (26.21%)	10,398 (23.99%)		9280 (26.64%)	8364 (24.01%)	
Southern	11,779 (28.00%)	12,126 (27.97%)		9680 (27.79%)	9640 (27.68%)	
Eastern	1288 (3.06%)	1013 (2.34%)		986 (2.83%)	832 (2.39%)	
Offshore islands	328 (0.78%)	100 (0.23%)		187 (0.54%)	59 (0.17%)	
Baseline healthcare use						
OPD visits (mean)	40.3	36.0	< .0001***	37.9	38.3	0.0692
Admission (N, %)			< .0001***			< .0001***
No	23,778 (56.52%)	31,432 (72.51%)		22,473 (64.52%)	23,464 (67.37%)	
Yes	18,292 (43.48%)	11,915 (27.49%)		12,356 (35.48%)	11,365 (32.63%)	
Previous year healthcare use						
OPD visits (mean, SD)^b^	37.7 ± 25.7	35.0 ± 24.0	< .0001***	36.1 ± 24.3	36.6 ± 24.9	0.0034**
Admission (N, %)^b^			< .0001***			< .0001***
No	27,342 (64.99%)	31,722 (73.18%)		23,727 (68.12%)	25,131 (72.16%)	
Yes	14,728 (35.01%)	11,625 (26.82%)		11,102 (31.88%)	9698 (27.84%)	
Facility characteristics						
Hospital accreditation level (N, %)			< .0001***			0.7107
Medical center	10,511 (25.56%)	12,152 (28.03%)		9381 (26.93%)	9405 (27.00%)	
Regional hospital	15,019 (36.53%)	15,119 (34.88%)		12,649 (36.32%)	12,603 (36.19%)	
District hospital	8012 (19.48%)	6399 (14.76%)		5802 (16.66%)	5901 (16.94%)	
Clinic	7577 (18.43%)	9332 (21.53%)		6997 (20.09%)	6920 (19.87%)	
Hospital Ownership (N, %)			< .0001***			0.0773
Public	16,487 (39.19%)	15,271 (35.23%)		13,031 (37.41%)	13,257 (38.06%)	
Private	25,583 (60.81%)	28,076 (64.77%)		21,798 (62.59%)	21,572 (61.94%)	

*COCI* Continuity of care index, *CCI* Charlson Comorbidity Index, *OPD* Outpatient department, *ref* Reference group. ^a^Low COCI: COCI < 1; High COCI: COCI = 1. ^b^indicates that the variable was not included in the calculation of the propensity score. *p-value < 0.05; **p-value < 0.01; ***p-value < 0.0001

### All-cause and potentially avoidable hospitalizations

Table 2 shows the results from logistic regression models of COCI on all-cause hospitalizations in patients with dementia. After controlling for the status of the patients, healthcare use in the previous year, and facility characteristics, the odds of hospitalization in the high COCI group were 0.848 times that of the low COCI group (95% CI:0.821–0.875), showing that people with high COCI are less likely to be hospitalized. Improving the continuity of care for patients with dementia would reduce the odds of hospitalization by 15.2%. Regarding the characteristics of patients with dementia, the odds ratio of hospitalization increased with age (OR = 1.035, 95% CI: 1.033–1.037), male gender (OR 1.273, 95% CI: 1.233–1.314), higher CCI score (OR = 1.153, 95% CI: 1.105–1.202), and low-income household status (OR = 1.38, 95% CI: 1.164–1.636).

**Table 2:** Logistic regression models of continuity of care on all-cause hospitalizations in patients with dementia

Variables	All-cause admission (*n* = 69,658)	
	OR	95% CI
Patient characteristics		
High COCI (ref: Low COCI)^a^	0.848	(0.821 ~ 0.875)***
Age	1.035	(1.033 ~ 1.037)***
Male (ref: Female)	1.273	(1.233 ~ 1.314)***
CCI score (ref: 0)		
≧1	1.153	(1.105 ~ 1.202)***
Low income household status (ref: No)	1.380	(1.164 ~ 1.636)**
Residence location (ref: North)		
Central	1.129	(1.084 ~ 1.175)***
Southern	1.121	(1.078 ~ 1.165)***
Eastern	1.424	(1.291 ~ 1.571)***
Offshore	0.638	(0.476 ~ 0.855)**
Previous year healthcare use		
OPD visits	1.004	(1.003 ~ 1.004)***
Admission (ref: No admission)	1.697	(1.638 ~ 1.759)***
Facility characteristics		
Hospital accreditation level (ref: Medical center)		
Regional	1.123	(1.079 ~ 1.170)***
District	1.187	(1.130 ~ 1.247)***
Clinic	0.967	(0.922 ~ 1.015)
Hospital Ownership (ref: Public)		
Private	1.037	(1.003 ~ 1.072)*

*COCI* Continuity of care index, *CCI* Charlson Comorbidity Index, *OPD* Outpatient department, *OR* Odds ratio, *CI* Confidence interval, *ref* Reference group. ^a^Low COCI: COCI < 1; High COCI: COCI = 1. *p-value < 0.05; **p-value < 0.01; ***p-value < 0.0001

Table 3 shows the association between COCI and PAHs in patients with dementia based on logistic regression models while controlling for various patient and facility characteristics. Results shown here were based on 4 disease-specific samples, and the number of patients with the specific disease used in each PAH model was shown respectively in the row heading of the table. There was no significant association found between COCI and PAHs among patients with dementia across the five disease outcomes. Compared to female patients with dementia, males had significantly higher risks of PAHs for DM long-term complications (OR = 1.21; 95% CI: 1.06–1.39), and COPD/asthma (OR = 1.54; 95% CI: 1.32 ~ 1.79). The risks of PAHs increased significantly with the number of comorbidities for DM long-term complications; the odds ratios for those with CCI scores being equal or more than 2 compared to those with CCI score being zero were 2.49 (95% CI: 1.61–3.85) Compared to those without admissions in the previous year, the odds ratios of those with prior admissions were much higher for DM short-term complications (OR = 1.41, 95% CI: 1.12 ~ 1.79), DM long-term complications (OR = 1.73, 95% CI: 1.51 ~ 1.99), COPD/asthma (OR = 2.24; 95% CI: 1.91–2.63), and heart failure (OR = 1.48, 95% CI: 1.02–1.05).

**Table 3:** Logistic regression models of continuity of care on potentially avoidable hospitalizations (PAH) in patients with dementia

Variables	DM short-term complications (*n* = 19,702)		DM long-term complications (*n* = 19,702)		Hypertension (*n* = 36,624)		COPD/Asthma (*n* = 7,602)		Heart failure (*n* = 5,606)	
	OR	95% CI	OR	95% CI	OR	95% CI	OR	95% CI	OR	95% CI
Patient characteristics										
High COCI (ref: Low COCI)^a^	0.846	(0.676–1.059)	0.930	(0.814 ~ 1.062)	0.819	(0.399 ~ 1.684)	0.986	(0.851 ~ 1.143)	1.063	(0.912 ~ 1.239)
Age	1.017	(1.000 ~ 1.034)	0.999	(0.989 ~ 1.009)	1.007	(0.954 ~ 1.064)	1.035	(1.024 ~ 1.047)	1.034	(1.022 ~ 1.046)
Male (ref: Female)	0.991	(0.787 ~ 1.247)	1.213	(1.060 ~ 1.388)	0.569	(0.251 ~ 1.290)	1.536	(1.320 ~ 1.787)	1.080	(0.923 ~ 1.263)
CCI score										
0	1.000			1.000						
1	1.379	(0.744 ~ 2.557)	1.105	(0.697 ~ 1.752)	0.675	(0.259 ~ 1.763)	1.000		1.000	
≧2	1.776	(0.974 ~ 3.240)	2.488	(1.608 ~ 3.850)	0.827	(0.328 ~ 2.086)	0.852	(0.713 ~ 1.018)	1.085	(0.874 ~ 1.347)
Residence location (ref: North)										
Central	1.068	(0.806 ~ 1.416)	1.017	(0.862 ~ 1.200)	0.979	(0.399 ~ 2.402)	1.129	(0.939 ~ 1.358)	1.092	(0.901 ~ 1.324)
Southern	1.028	(0.779 ~ 1.355)	0.826	(0.699 ~ 0.977)	0.666	(0.251 ~ 1.769)	0.927	(0.768 ~ 1.118)	1.136	(0.934 ~ 1.383)
Eastern/offshore	1.744	(1.012 ~ 3.006)	0.834	(0.549 ~ 1.266)	3.065	(0.863 ~ 10.882)	1.520	(1.068 ~ 2.164)	1.621	(1.133 ~ 2.319)
Previous year healthcare use										
OPD visits	0.995	(0.990 ~ 1.000)	1.004	(1.001 ~ 1.006)	1.010	(0.999 ~ 1.021)	1.002	(0.999 ~ 1.004)	1.003	(1.000 ~ 1.005)
Admission (ref: No admission)	1.412	(1.115 ~ 1.787)	1.733	(1.509 ~ 1.990)	0.373	(0.146 ~ 0.950)	2.237	(1.905 ~ 2.626)	1.475	(1.250 ~ 1.740)
Facility characteristics										
Hospital accreditation level (ref: Medical center)										
Regional	1.329	(0.992 ~ 1.780)	1.193	(1.005 ~ 1.416)	1.971	(0.698 ~ 5.566)	1.139	(0.929 ~ 1.396)	1.15	(0.947 ~ 1.395)
District	1.171	(0.816 ~ 1.680)	1.482	(1.218 ~ 1.803)	1.478	(0.423 ~ 5.166)	1.478	(1.188 ~ 1.838)	0.983	(1.778 ~ 1.242)
Clinic	1.321	(0.928 ~ 1.881)	0.839	(0.667 ~ 1.055)	1.501	(0.466 ~ 4.828)	0.924	(0.720 ~ 1.186)	0.662	(0.503 ~ 0.872)
Hospital Ownership (ref: Public)										
Private	1.409	(1.099 ~ 1.805)	0.871	(0.758 ~ 0.999)	1.681	(0.738 ~ 3.825)	1.181	(1.012 ~ 1.380)	1.014	(0.865 ~ 1.189)

*COCI* Continuity of care index, *CCI* Charlson Comorbidity Index, *OPD* Outpatient department, *DM* Diabetes, *COPD* Chronic obstructive pulmonary disease, *OR* Odds ratio, *CI* Confidence interval, *ref* Reference group. ^a^Low COCI: COCI < 1; High COCI: COCI = 1

### Healthcare costs

Table 4 presents the regression results of the impact of COCI on outpatient, hospitalization, and total healthcare costs in patients with dementia. We constructed three separate analyzed samples for each type of cost by excluding outliers 3 standard deviations from their respective mean value. The analytic sample for outpatient costs included 68,543 people, and results showed that the outpatient costs of the high COCI group were 4% lower than that of the low COCI group after controlling for patient and facility characteristics and prior healthcare use. The analytic sample for hospitalization costs was 67,130 people, and the hospitalization costs of the high COCI group were 33.7% lower than that of the low COCI group after controlling for all other variables. The analytic sample for total healthcare costs was 66,677 people, and after controlling for all other variables, the total healthcare costs of the high COCI group were 3.8% lower than that of the low COCI group.

**Table 4:** Generalized linear models of continuity of care on outpatient, hospitalization, and total healthcare costs in patients with dementia

Variables	Outpatient costs (*N* = 68,543)		Hospitalization costs (*N* = 67,130)		`Total costs (*N* = 66,677)	
	exp(β)	95% CI	exp(β)	95% CI	exp(β)	95% CI
Patient characteristics						
High COCI (ref: Low COCI)^a^	0.960	(0.941 ~ 0.979)***	0.663	(0.614 ~ 0.717)***	0.962	(0.945 ~ 0.980)***
Age	0.984	(0.982 ~ 0.985)***	1.088	(1.082 ~ 1.094)***	0.985	(0.983 ~ 0.986)***
Male (ref: Female)	0.983	(0.963 ~ 1.003)	1.815	(1.677 ~ 1.964)***	0.988	(0.969 ~ 1.007)
CCI Score (ref: 0)						
1	1.155	(1.125 ~ 1.185)***	1.323	(1.197 ~ 1.462)***	1.162	(1.134 ~ 1.191)***
≧2	1.269	(1.236 ~ 1.305)***	2.488	(2.239 ~ 2.762)***	1.287	(1.255 ~ 1.320)***
Low income household status (ref: No)	0.838	(0.751 ~ 0.934)**	2.387	(1.557 ~ 3.658)***	0.872	(0.787 ~ 0.967)**
Residence location (ref: North)						
Central	0.989	(0.965 ~ 1.014)	1.321	(1.197 ~ 1.459)***	0.999	(0.975 ~ 1.023)
Southern	0.948	(0.925 ~ 0.971)***	1.340	(1.218 ~ 1.474)***	0.959	(0.938 ~ 0.982)***
Eastern	1.058	(0.992 ~ 1.127)	2.597	(2.026 ~ 3.330)***	1.056	(0.994 ~ 1.121)
Offshore islands	0.773	(0.653 ~ 0.916)**	0.380	(0.198 ~ 0.731)**	0.783	(0.669 ~ 0.917)**
Previous year healthcare use						
OPD visits	1.015	(1.015 ~ 1.016)***	1.009	(1.007 ~ 1.010)***	1.016	(1.015 ~ 1.016)***
Admission	0.951	(0.929 ~ 0.973)***	3.881	(3.543 ~ 4.250)***	0.976	(0.955 ~ 0.997)*
Facility characteristics						
Hospital accreditation level (ref: Medical center)						
Regional	0.861	(0.839 ~ 0.882)***	1.303	(1.179 ~ 1.439)***	0.861	(0.841 ~ 0.882)***
District	0.824	(0.798 ~ 0.850)***	1.534	(1.357 ~ 1.733)***	0.840	(0.816 ~ 0.866)***
Clinic	0.754	(0.731 ~ 0.776)***	0.952	(0.847 ~ 1.070)	0.752	(0.731 ~ 0.773)***
Hospital Ownership (ref: Public)						
Private	1.071	(1.048 ~ 1.093)***	1.055	(0.972 ~ 1.145)	1.065	(1.044 ~ 1.085)***

*COCI* Continuity of care index, *CCI* Charlson Comorbidity Index, *OPD* Outpatient department, *DM* Diabetes, *COPD* Chronic obstructive pulmonary disease, *CI* Confidence interval, *ref* Reference group. ^a^Low COCI: COCI < 1; High COCI: COCI = 1

Looking at the tables of healthcare costs, the columns on the left showed other factors that were found to increase the outpatient costs of patients with dementia, including higher CCI scores, more outpatient visits in the previous year, and private ownership of the most frequently visited healthcare facility. The columns in the middle showed other factors that were found to increase the hospitalization costs of patients with dementia, including older age, male gender, higher CCI scores, more outpatient visits or admissions in the previous year, and the most- frequently visited healthcare facility being a regional or district hospital. The columns on the right showed other factors that increased the total healthcare costs of patients with dementia, including higher CCI scores, more outpatient visits in the previous year, and private ownership of the most frequently visited healthcare facility.

## Discussion

The continuity of care for patients with dementia in Taiwan had a mean COCI of 0.74 and a standard deviation of 0.3, with more than half of the patients having a COCI score of 1, which means that they had good physician continuity. Research has pointed out that low levels of COC have been associated with higher rates of hospitalizations and healthcare costs in the dementia population [21]. Amjad et al. reported that the annual hospitalization rate per beneficiary was 5.8% higher in the lowest continuity group in contrast to the highest continuity group [21]. Our finding, which showed that a higher continuity of care could reduce the odds of hospitalization by 15.2% in patients with dementia, was consistent with previous research. It means that having a regular physician in dementia care can prevent the hospitalization of patients with dementia.

In this study, there was no statistically significant correlation between dementia-related COCI and any types of PAH measured by MACIEs after controlling for other variables. This finding was different from previous studies that examined the effect of disease-specific COC such as patients with diabetes or COPD. One study found that diabetic patients with low to medium continuity of care were significantly associated with increased risk of long-term diabetic complications and lower extremity amputations [33]. Another two studies were on patients with COPD and both showed that after controlling for covariates, subjects in the low COCI group were more likely (adjusted odds ratio being greater than 2) to undergo COPD-related avoidable hospitalizations than those in the high COCI group [34, 35]. Similarly, older adults with dementia and low COC were more likely to have dementia-related hospitalizations, instead of potentially avoidable hospitalization in general [24]. Nevertheless, our results are similar to two previous studies that showed better COC was not associated with a lower rate of ACSC hospitalization among patients with dementia [21, 24]. One reason discussed in the literature was that ACSC conditions may be overshadowed by delirium, and those early symptoms may be missed even with high physician continuity among older patients with dementia [21]. In other words, the concept of ACSC or “potentially avoidable” conditions was applied to all outpatient care recipients, and not specifically older adults with dementia [12]. Our finding also echoed with an earlier study suggesting a need to define preventable hospitalization specifically for patients with dementia due to their reduced ability to self-manage chronic conditions [24].

 Regarding healthcare costs, the results of this study showed that when patients with dementia have better continuity of care, it can reduce medical expenditures across all aspects, including outpatient costs by 4%, hospitalization costs by 33.7%, and total costs by 3.8%. Using a longitudinal cohort design with propensity score matching to control for confounding, our finding is consistent with two previous studies that showed that continuity of care could reduce total medical expenditures incurred by lowering hospitalization [21, 26]. Patients with dementia are at risk of experiencing in-hospital adverse events during medical care [36]. This population is also at risk of unnecessary lab testing, leading to physical and financial burden through invasive medical tests and overtreatment [21]. Due to the associated adverse outcomes and related costs, the focus of dementia care has been on improving quality for the past two decades. Previous research has emphasized the importance of COC for patients with dementia due to the long duration of the disease, which requires ongoing knowledge of patients’ medical and psychosocial conditions [26]. As our study showed that higher continuity in dementia-related outpatient visits was associated with lower healthcare costs in all aspects, improving the continuity of outpatient care for patients with dementia will inevitably reduce subsequent hospitalization and save medical costs.

One crucial difference between our study and the literature was the method for calculating continuity of care. We calculated COCI according to the type of outpatient visit while in the study by Amjad et al. [21], the care of continuity was calculated based on the records of all outpatient visits that year, so it was not limited to the disease of dementia. But if there were more comorbidities, the lower the continuity score would become. Thus, the lower continuity of care score could not accurately reflect the patients’ continuity of care. It was more challenging to prove the effectiveness of the continuity of care for patients with dementia by using all outpatient visits. Other studies that examined the continuity of care among patients with dementia previously have either limited their calculation to primary care providers [9] or by only counting visits to both primary care providers and dementia-related specialists (neurologist, psychiatrist, psychologist, and social worker) [24, 26], so their definition was different from our definition of dementia-related continuity of care.

This study has several strengths. First, by restricting the measurement of COCI to include physician visits with dementia diagnoses, the contribution of the current study is that we aimed to evaluate the impact of dementia-related continuity of care on all-cause hospitalization, PAHs, and healthcare costs focusing on the effectiveness of dementia-related outpatient visits which has not been done before. Second, the data were obtained from the Taiwan National Health Insurance Research Database, which contains information from nationally representative data, and our cohort study design was superior to past cross-sectional studies on patients with dementia.

Several limitations need to be considered while interpreting our study. Several limitations need to be considered while interpreting our study. First, even though our research used claims-based dementia diagnoses, this may under-represent the whole dementia population since those with mild dementia would not have been diagnosed nor been included in our sample. Moreover, our claims data did not include measures on dementia severity, so we used the CCI scores and healthcare utilization records from the previous year as a proxy for disease severity. Second, the exclusion criteria of patients who died during the year, or resided in nursing care facilities, or with less than 3 outpatient visits were applied to make COCI computable, but that also limited the generalizability of our findings to populations with regular physician contact. Third, although we used propensity score matching to reduce selection bias, unmeasured confounding factors might still affect our results [37]. For example, our claims data did not include information on socioeconomic status or caregiver support of patients with dementia. Therefore, the relationship between the continuity of care and the outcome of hospitalization could be biased because informal caregivers might be more involved in communicating with physicians and arranging health services for patients when their dementia becomes more severe. Fourth, this study used five MACIE indicators as the outcome measure for PAH in order to compare with existing literature on older adults with dementia, but in our supplemental analysis of all-cause hospitalization, urinary tract disease was found to be the most common cause of hospitalization; therefore, future studies may also consider using other indicators, such as the PQIs which contain 16 ACSCs including urinary tract infections to elucidate possible correlations between COCI and avoidable hospitalization [23].

## Conclusion

Greater continuity of care for dementia-related outpatient visits was associated with a reduced likelihood of all-cause hospitalization and medical expenditures, but there was no significant effect on PAHs. However, this study demonstrated the many benefits of continuity of care, including reduced hospitalization and healthcare costs. Therefore, promoting continuity of care among individuals with dementia is recommended.

## Data Availability

The data that support the findings of this study are available from the Health and Welfare Data Science Center (HWDC) of Taiwan, but restrictions apply to the availability of these data, which were applied to be used exclusively for the current study, and so are not publicly available.

## References

[1] Dementia [https://www.who.int/news-room/fact-sheets/detail/dementia.]

[2] Fratiglioni L (2000). The transition from normal functioning to dementia in the aging population. Neurobiol Aging.

[3] Dementia: A public health priority [https://apps.who.int/iris/handle/10665/75263]

[4] Timmons S, Manning E, Barrett A, Brady NM, Browne V, O’Shea E, Molloy DW, O'Regan NA, Trawley S, Cahill S (2015). Dementia in older people admitted to hospital: a regional multi-hospital observational study of prevalence, associations and case recognition. Age Ageing.

[5] Lyketsos CG, Sheppard JM, Rabins PV (2000). Dementia in elderly persons in a general hospital. Am J Psychiatry.

[6] Zhu CW, Cosentino S, Ornstein K, Gu Y, Andrews H, Stern Y (2015). Use and cost of hospitalization in dementia: Longitudinal results from a community-based study. Int J Geriatr Psychiatry.

[7] Bynum JPW, Rabins PV, Weller W, Niefeld M, Anderson GF, Wu AW (2004). The relationship between a dementia diagnosis, chronic illness, medicare expenditures, and hospital use. J Am Geriatr Soc.

[8] Hsiao FY, Peng LN, Wen YW, Liang CK, Wang PN, Chen LK (2015). Care needs and clinical outcomes of older people with dementia: A population-based propensity score-matched cohort study. PLoS ONE.

[9] Godard-Sebillotte C, Strumpf E, Sourial N, Rochette L, Pelletier E, Vedel I (2021). Primary care continuity and potentially avoidable hospitalization in persons with dementia. J Am Geriatr Soc.

[10] Feng Z, Coots LA, Kaganova Y, Wiener JM (2014). Hospital and ed use among Medicare beneficiaries with dementia varies by setting and proximity to death. Health Aff.

[11] Toseef M, Jensen Summers G, Tarraf W (2018). Effects of medicaid health maintenance organizations on potentially preventable hospitalizations. Innov Aging.

[12] Phelan EA, Borson S, Grothaus L, Balch S, Larson EB (2012). Association of incident dementia with hospitalizations. JAMA.

[13] Huang Y, Meyer P, Jin L (2019). Spatial access to health care and elderly ambulatory care sensitive hospitalizations. Public Health.

[14] Rocha JVM, Santana R, Tello JE (2021). Hospitalization for ambulatory care sensitive conditions: What conditions make inter-country comparisons possible?. Health Policy OPEN.

[15] AHRQ Quality Indicators—Guide to Prevention Quality Indicators: Hospital Admission for Ambulatory Care Sensitive Conditions. Rockville, MD: Agency for Healthcare Research and Quality, 2001. AHRQ Pub. No. 02-R0203. https://ahrq.gov/downloads/pub/ahrqqi/pqiguide.pdf.

[16] Westrick E (2006). Medicare ambulatory care indicators for the elderly [electronic resource] : refinement of the Access to Care for the Elderly Project indicators : final report / Edward Westrick, Stephen Kogut.

[17] Lin P-J, Zhong Y, Fillit HM, Cohen JT, Neumann PJ (2017). Hospitalizations for ambulatory care sensitive conditions and unplanned readmissions among Medicare beneficiaries with Alzheimer's disease. Alzheimers Dement.

[18] Desai U, Kirson NY, Ye W, Mehta NR, Wen J, Andrews JS (2019). Trends in health service use and potentially avoidable hospitalizations before Alzheimer's disease diagnosis: A matched, retrospective study of US Medicare beneficiaries. Alzheimer's & Dementia: Diagnosis, Assessment & Disease Monitoring.

[19] National Health Insurance Administration (2017). National Health Insurance Annual Report 2017–2018. Ministry of Health and Welfare, Taiwan.

[20] Chen CC, Tseng CH, Cheng SH (2013). Continuity of care, medication adherence, and health care outcomes among patients with newly diagnosed type 2 diabetes: a longitudinal analysis. Med Care.

[21] Amjad H, Carmichael D, Austin AM, Chang C-H, Bynum JPW (2016). Continuity of care and health care utilization in older adults with dementia in fee-for-service medicare. JAMA Intern Med.

[22] Kao YH, Lin WT, Chen WH, Wu SC, Tseng TS (2019). Continuity of outpatient care and avoidable hospitalization: a systematic review. Am J Manag Care.

[23] Wolf D, Rhein C, Geschke K, Fellgiebel A (2019). Preventable hospitalizations among older patients with cognitive impairments and dementia. Int Psychogeriatr.

[24] Lei L, Cai S, Conwell Y, Fortinsky RH, Intrator O. Can continuity of care reduce hospitalization among community-dwelling older adult veterans living with dementia? Medical Care. 2020;58(11):988-995. 10.1097/MLR.0000000000001386 32925470

[25] Hussey PS, Schneider EC, Rudin RS, Fox DS, Lai J, Pollack CE (2014). Continuity and the costs of care for chronic disease. JAMA Intern Med.

[26] Lei L, Intrator O, Conwell Y, Fortinsky RH, Cai S (2021). Continuity of care and health care cost among community-dwelling older adult veterans living with dementia. Health Serv Res.

[27] Chen CC, Cheng SH (2016). Continuity of Care and Changes in Medication Adherence Among Patients With Newly Diagnosed Diabetes. Am J Manag Care.

[28] Maxwell CJ, Amuah JE, Hogan DB, Cepoiu-Martin M, Gruneir A, Patten SB, Soo A, Le Clair K, Wilson K, Hagen B (2015). Elevated hospitalization risk of assisted living residents with dementia in Alberta, Canada. J Am Med Dir Assoc.

[29] Johnston KJ, Hockenberry JM (2017). Patient continuity of care and physician management of older adults with complex chronic conditions. Innov Aging.

[30] Hussein A, Carrière KC (2002). A measure of continuity of care based on the multiplicative intensity model. Stat Med.

[31] Bice TW, Boxerman SB. A quantitative measure of continuity of care. Medical Care. 1977;15(4):347-349. 10.1097/00005650-197704000-00010 859364

[32] Charlson ME, Pompei P, Ales KL, MacKenzie CR (1987). A new method of classifying prognostic comorbidity in longitudinal studies: Development and validation. J Chronic Dis.

[33] Lin W, Huang I-C, Wang S-L, Yang M-C, Yaung C-L (2009). Continuity of diabetes care is associated with avoidable hospitalizations: evidence from Taiwan's National Health Insurance scheme. Int J Qual Health Care.

[34] Lin IP, Wu SC (2017). Effects of long-term high continuity of care on avoidable hospitalizations of chronic obstructive pulmonary disease patients. Health Policy.

[35] Lin IP, Wu SC, Huang ST (2015). continuity of care and avoidable hospitalizations for chronic obstructive pulmonary disease (COPD). J Am Board Fam Med.

[36] Mitsutake S, Ishizaki T, Tsuchiya-Ito R, Furuta K, Hatakeyama A, Sugiyama M, Toba K, Ito H (2021). Association of cognitive impairment severity with potentially avoidable readmissions: A retrospective cohort study of 8897 older patients. Alzheimer's & Dementia: Diagnosis, Assessment & Disease Monitoring.

[37] Austin PC (2011). An introduction to propensity score methods for reducing the effects of confounding in observational studies. Multivar Behav Res.

